# A theory of demographic optimality in forests

**DOI:** 10.1038/s41598-023-44860-7

**Published:** 2023-10-31

**Authors:** Jon Moore, Arthur Argles, Peter Cox

**Affiliations:** 1 Faculty of Environment, Science and Economy, University of Exeter, Exeter, Devon EX4 4QF UK; 2 Met Office Hadley Centre, Fitzroy Road, Exeter, EX1 3PB UK

**Keywords:** Theoretical ecology, Climate-change ecology, Ecological modelling, Forest ecology

## Abstract

Carbon uptake by the land is a key determinant of future climate change. Unfortunately, Dynamic Global Vegetation Models have many unknown internal parameters which leads to significant uncertainty in projections of the future land carbon sink. By contrast, observed forest inventories in both Amazonia and the USA show strikingly common tree-size distributions, pointing to a simpler modelling paradigm. The curvature of these size-distributions is related to the ratio of mortality to growth in Demographic Equilibrium Theory (DET). We extend DET to include recruitment limited by competitive exclusion from existing trees. From this, we find simultaneous maxima of tree density and biomass in terms of respectively the ratio of mortality to growth and the proportion of primary productivity allocated to reproduction, an idea we call Demographic Optimality (DO). Combining DO with the ratio of mortality to growth common to the US and Amazon forests, results in the prediction that about an eighth of productivity should be allocated to reproduction, which is broadly consistent with observations. Another prediction of the model is that seed mortality should decrease with increasing seed size, such that the advantage of having many small seeds is nullified by the higher seed mortality. Demographic Optimality is therefore consistent with the common shape of tree-size distributions seen in very different forests, and an allocation to reproduction that is independent of seed size.

## Introduction

Predicting how forests respond to climate and land-use change is of critical importance in climate research. There is still significant uncertainty in the predictions of what proportion of human carbon emissions are taken in by ecosystems on land (global land carbon sink)^1^. This is, in part, due to the challenges of modelling the complexities of global vegetation, across large scales and multiple biomes^2–5^. To meet this challenge medium complexity dynamic global vegetation models (DGVMs), that find a balance between representing complex processes and yet being simple enough to be usable at a large scale, are increasingly becoming seen as a solution^6^.

Global forest models predict forest-level metrics, such as biomass density, tree density or canopy coverage based on tree traits, scaling laws from allometry and modelling of processes. The values of the traits in these models are derived in various ways, including from observed data and the understanding of physiological processes. Some of these traits have greater uncertainty in their values or have only sparse observed data. An interesting question is what underlying principles lead to the observed traits and do these traits lead to observed size-distributions of trees in forests?

Three key drivers of forest dynamics are growth, mortality and reproduction. Combined with the scaling of tree dimensions with size (allometry) these determine the ultimate forest-level metrics of a forest as it reaches maturity. Given these drivers, the tree-size distribution can be modelled using the principle of continuity. Which implies that the numbers of trees in any size range is determined by the number of trees growing into that size range from below, the number trees growing out to larger size classes, and the loss of trees due to mortality. When applied to forests close to equilibrium (unchanging size-structure) we get the theory known as Demographic Equilibrium Theory (DET)^7^.

**Figure 1:**
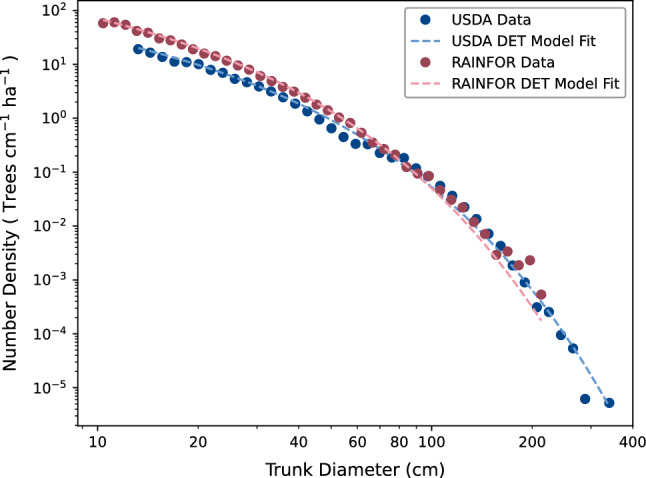
Evidence of similar ratios of mortality to growth in forest worldwide. Blue dots show binned observations for North American trees using observations of trunk diameter (diameter at breast height) from the USDA Forest Service FIA program, and red dots show likewise for the RAINFOR measurements of basal diameter in Amazonia^21^. The best fit DET profiles are shown by the red dashed line and the light-blue dashed line, respectively. Figure based on^6^, using data from^8,9^.

Using DET we have previously shown that there are very similar shapes of forest size-distribution in both the US^8^ and Amazon^9^. These studies used the fact that DET predicts a size-distribution known as the Weibull distribution. We used a form of the distribution that allowed us to characterise the shape of the distribution purely through a single parameter that represents the ratio of mortality to growth at a reference tree size. While the value of this parameter varies at local scales, a similar common value emerges when combining multiple sites across the very different forests of North America and Amazonia (Fig. 1).

The third driver of forest dynamics is reproduction, which is not previously directly modelled in DET. Forests allocate part of their productivity to reproduction so as to have enough seeds to survive through to adulthood. The literature is somewhat sparse as to how much of the productivity of a forest is allocated to reproduction, but of the few studies available it is suggested that 5–15% of net primary productivity (NPP) is allocated to reproduction^10^ or 3% and 20% of annual gross primary production (GPP)^11^.

**Figure 2:**
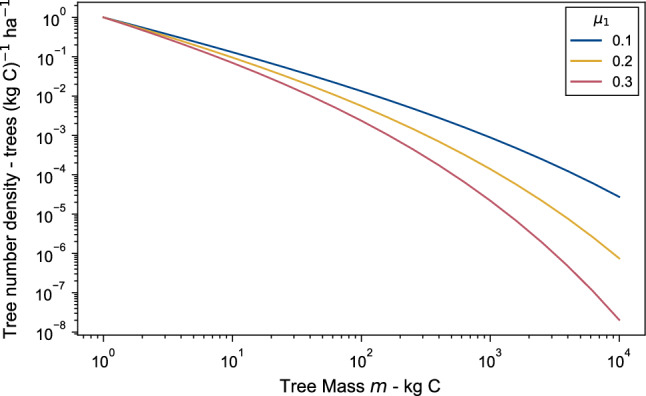
The effect of the mortality to growth ratio for a 1 kg C size tree $\mu _1$ on the curvature of a forest size distribution. Increasing $\mu _1$ means mortality rate is increasing relative to the growth rate and so more trees die before they grow as big.

**Figure 3:**
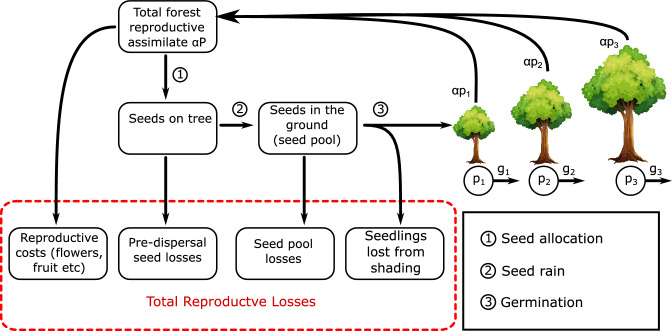
Reproductive fluxes in the model. Adult trees all have a net assimilate *p* (NPP - Litter losses) and allocate a proportion of $\alpha$ of *p* to reproduction and the remainder to growth *g*. Larger trees have a greater assimlate and hence growth and reproductive flux. Reproduction comes with costs such as flowers, fruit, seed cases etc, the remaining reproductive allocation becomes seeds (1). Seeds then can be lost off the tree due to processes such as herbivory before raining down on the ground (2). Seeds can suffer further mortality on the ground before germination (3). Final losses due to competitive suppression and shading, with the remaining flux joining the forest size distrubution. The parameter $f_{rs}$ represents the fraction of reproduction flux reaching (3) before the shading losses, so the flux at (3) is $\alpha f_{rs} P$.

The distribution of trees across size is therefore dependent on the rate of growth, mortality and reproduction. This raises the question of whether optimality can be seen in forest models and whether the common mortality-to-growth ratio and observed allocation to reproduction are related. Are the mean values of these two traits the result of an emergent property resulting from the evolution, competition and physical limits of physiological processes? Do these processes in turn maximise the forest-level properties like biomass density and tree density?

This study will investigate the question of whether there are particular values of the parameters of the DET equilibrium solutions that can lead to an maxima in the forest-wide properties such as biomass, and total tree density. It will also investigate how any such optima may relate to the previously observed similar values of mortality to growth ratio across the US and Amazon.

## Demographic Equilibrium Theory

The forest model used in this study, known as Demographic Equilibrium Theory^7–9^ (DET), describes the distribution of tree sizes in a forest that has reached a steady state under constant environmental conditions and no stochastic disturbances. To keep the model analytically tractable we also currently assume no under-storey, nor stratification of forest layers and that we have just one tree type that is the average of many competing species seen in a real forest. In this model the number of trees in any given size range is simply determined by the balance of smaller trees growing into that size range and the trees leaving the size range through growth and mortality. This process is described by the one-dimensional drift or continuity equation (Eq. 1) with an added loss term for mortality^12^. This equation can also be obtained from the Kolmogorov forward or the Fokker-Planck equation if the second-order term is omitted^13^:

1 $$\begin{aligned} \frac{\partial n(m,t)}{\partial t} + \frac{\partial }{\partial m}\left( n(m,t) \, g(m,t) \right) = - \gamma (m,t) \, n(m,t), \end{aligned}$$

where *n* is the size distribution (tree density per size class) in trees per unit tree mass per unit area, in terms of tree mass, *m*, tree mass growth rate, *g*, and mortality rate, $\gamma$, over time, *t*.

The demographic equilibrium size distribution can be obtained from Eq. (1) through integration of Eq.^12,14^:

2 $$\begin{aligned} \int _{n_s^{}}^n \dfrac{d\tilde{n}}{\tilde{n}} = \int _{m_s^{}}^m - \dfrac{1}{g(\tilde{m})} \left[ \dfrac{dg}{d\tilde{m}} + \gamma \right] d\tilde{m}, \end{aligned}$$

where the lower bound of the integration corresponds to seedlings of mass $m_s^{}$ and density $n_s$. For DET the mass growth rate is assumed to scale as a power law, (Eq. 3) according to Metabolic Scaling Theory^15,16^ (MST):

3 $$\begin{aligned} g(m) = g_r \left( \dfrac{m}{m_r} \right) ^{3/4}, \end{aligned}$$

where $g_r$ is the tree growth rate (in kg C $\hbox {yr}^{-1}$) at some fixed tree size, $m_r$ (kg C), and the scaling power 3/4 is derived from MST. To keep the equations tractable the mortality, $\gamma$, is assumed to be size-invariant. We believe this is an acceptable compromise as growth scaling seems to matter more in these type of models^17^ and has previously been fitted to tree inventory data successfully^8,9^.

The integration results in a left-truncated Weibull size distribution^7,9^:

4 $$\begin{aligned} n(m) = n_r \left( \dfrac{m}{m_r} \right) ^{-3/4} \exp \left( 4 \mu _r \left\{ 1 - \left( \dfrac{m}{m_r} \right) ^{1/4} \right\} \right) , \end{aligned}$$

where $n_r$ is the tree density function, *n* (trees per $\hbox {m}^2$ per kg C), at a reference tree size; $m_r$ (useful choices for this could be the seedling size $m_s$ or $m_1 = 1$ kg C). The dimensionless quantity $\mu _r$ is the ratio of the rate biomass loss due to mortality to the rate of biomass gain from growth for a tree of mass $m_r$, defined mathematically as:

5 $$\begin{aligned} \mu _r = \dfrac{\gamma m_r}{g_r}. \end{aligned}$$

The $\mu _r$ parameter affects the curvature of the Weibull size distribution, with a larger value meaning the mortality is higher relative to the growth and so more trees are lost as any particular cohort of trees of a given size grows (Fig. 2). So a high $\mu _r$ forest will have a much larger proportion of small trees compared to a low $\mu _r$ forest.

Despite the simplifying assumptions, Eq. (4) has been validated against observations across a large scale and many sites for tropical forests^7,9,17,18^ and the US^8^.

Equation (4) can in turn be integrated^9,19^ to obtain the respective equations for the equilibrium total forest tree density, *N* (trees per $\hbox {m}^2$), total biomass density, *M* (kg C per $\hbox {m}^2$):

6 $$\begin{aligned} N= & {} \int _{m_s^{}}^{\infty } n(m) \, dm = \dfrac{n_s g_s}{\gamma }, \end{aligned}$$

7 $$\begin{aligned} M= & {} \int _{m_s^{}}^{\infty } m \, n(m) \, dm = N \bar{m}, \end{aligned}$$

where $n_s$ and $g_s$ are respectively the tree density function, *n*, and the growth at the seedling size, $m_s$, and $\bar{m} = M / N$ is the mean tree mass.

If we again use the assumption of MST power law allometry for tree crown area^20^, *a* (in $\hbox {m}^2$):

8 $$\begin{aligned} a(m) = a_r \left( \dfrac{m}{m_r} \right) ^{1/2}, \end{aligned}$$

then we can also integrate to obtain the fraction of ground area directly covered by tree crowns, called the fractional coverage, $\nu$:

9 $$\begin{aligned} \nu = \int _{m_s^{}}^{\infty } a(m) \, n(m) \, dm = N \bar{a}, \end{aligned}$$

where $\bar{a} = \nu / N$ is the mean tree crown area. The current version of this model assumes no overlap of tree crowns.

The mean mass, $\bar{m}$, and mean crown area, $\bar{a}$, evaluate to a finite series of terms involving $\mu _s$, which is the mortality to growth ratio of newly recruited seedlings of mass $m_s$ and crown area $a_s$:

10 $$\begin{aligned} \mu _s = \dfrac{\gamma m_s}{g_s}= \mu _r \left( \dfrac{m_s}{m_r} \right) ^{1/4}. \end{aligned}$$

The equations for the mean tree mass and crown area are respectively:

11 $$\begin{aligned} \bar{m}= & {} \dfrac{M}{N} = m_s \Big (1 + \dfrac{1}{\mu _s} + \dfrac{3}{4\mu _s^2} + \dfrac{3}{8\mu _s^3} + \dfrac{3}{32\mu _s^4} \Big ), \end{aligned}$$

12 $$\begin{aligned} \bar{a}= & {} \dfrac{\nu }{N} = a_s \Big (1 + \dfrac{1}{2\mu _s} + \dfrac{1}{8\mu _s^2} \Big ). \end{aligned}$$

The derivation of can be found in sections 1 and 2 of the supplementary material.

### Demographic Equilibrium Theory including reproduction

The theory so far only directly gives the relative abundance of any given size class (the shape of the equilibrium distribution) but will not give the absolute abundance of trees of any given size, as the scaling parameter $n_r$ (Eq. 4) is not explicitly specified in terms of the tree parameters. The reason for this is the lack of any modelling of the recruitment of tree seedlings. To have a complete theory, recruitment from existing trees within the size-structure needs to be included.

This can be done by assuming a fixed proportion, $\alpha$, of the NPP from photosynthesis left after litter losses (hereafter called assimilate, with *p* for tree assimilate and *P* for total forest assimilate) is allocated to reproduction with the remainder allocated to growth^19^: $g(m) = p(m) (1 - \alpha )$. Tree assimilate, *p*, is assumed to scale identically to growth:

13 $$\begin{aligned} p(m)= p_r \left( \dfrac{m}{m_r} \right) ^{3/4}. \end{aligned}$$

To satisfy equilibrium, the total number of seeds recruited in the forest is assumed to balance the total number of individuals lost through mortality. The rate of seeds produced by the forest per unit area is simply the total forest assimilate, *P*, multiplied by $\alpha$ and divided by the seed mass, $m_s$. We assume seeds and freshly germinated seedlings have the same mass. Losses of seeds due to various forms of mortality and reproduction costs (e.g., flowers, seed cases, fruit etc) are represented collectively by the fraction $f_{rs}$, which is the proportion of assimilate allocated to reproduction that survives to germination. It is assumed that the seeds recruited only survive if they germinate away from the shade of larger trees, so the recruitment is scaled by the amount of unshaded ground, which is one minus the total forest canopy coverage. Also, for simplicity, we assume MST allometry down to the seedling size. While this is unlikely to be realistic, the effect of any deviations are small as the contribution of such small seedlings to the overall forest biomass and coverage is small. This whole process is illustrated in Fig. 3 and is described by the boundary equation:

14 $$\begin{aligned} \dfrac{\alpha f_{rs} P (1- \nu )}{m_s} = N \gamma , \; \; \; 0 \le \nu \le 1. \end{aligned}$$

The total coverage, $\nu$, takes values between 0 and 1. This can simplified by noting that mean rate of seeds recruitment per tree, $\bar{s}$, is defined as:

15 $$\begin{aligned} \bar{s} = \dfrac{\alpha f_{rs} P}{ N m_s }, \end{aligned}$$

resulting in the boundary equation becoming:

16 $$\begin{aligned} \bar{s} (1 - \nu ) = \gamma , \end{aligned}$$

from which coverage, $\nu$, can be eliminated using Eq. (9). This can be rearranged to obtain an equation for the total tree density, *N*, in terms of mean tree crown area, $\bar{a}$, and mean tree rate of seed recruitment as well as tree mortality, $\gamma$:

17 $$\begin{aligned} N = \dfrac{1}{\bar{a}} \left( 1 - \dfrac{\gamma }{\bar{s}} \right) , \; \; \; \bar{s} > \gamma , \end{aligned}$$

which is a full equilibrium solution describing both the shape of the size distribution and the absolute abundance of trees of any given size. Note that if $\bar{s} < \gamma$, then this means there are not enough seeds produced to replace trees lost from mortality, so no forest can exist in equilibrium. By combining this with Eq. (7), the biomass is then:

18 $$\begin{aligned} M = \dfrac{\bar{m}}{\bar{a}} \left( 1 - \dfrac{\gamma }{\bar{s}} \right) , \; \; \; \bar{s} > \gamma . \end{aligned}$$

This model is the same as the equilibrium solution of the RED DGVM, except for the inclusion reproduction survival term $f_{rs}$. The inclusion of this term improves the model by collectively representing three processes of reproduction costs, these are seed production costs, pre-dispersal losses and post-dispersal / seed pool losses. For simplicity, these processes are combined to just give a single fractional value $f_{rs}$ in this study.

### Biomass and tree density maxima

Analytical equations have an advantage over numerical models, in that it is easy to explore the effects that key parameters have upon them. This allows for efficient investigation of whether there is an optimum value of forest-level properties such as biomass or tree density in terms of any given parameter.

For the remainder of this study, we will choose the reference tree mass $m_r$ to be equal to $m_1$=1 kg C, i.e. all size-dependent parameters will be defined in terms of a tree of mass 1 kg C. So we will define our tree trait parameters in terms of that, such as reference crown area $a_1$, growth $g_1$, assimilate $p_1$ and mortality to growth ratio $\mu _1$. Other parameters such as seed size, $m_s$, the proportion of assimilate allocated to reproduction, $\alpha$, and the proportion of reproductive assimilate reaching seed germination, $f_{rs}$, are assumed to be invariant with respect to tree size. While in reality $\alpha$ varies seasonally, and with tree size, in the context of this model we choose, for simplicity, for $\alpha$ to represent the mean reproductive effort of the forest.

One thing to note is that the reference size is chosen purely for convenience and to be consistent with previous work. As the study uses MST it is easy to translate any value to a larger size. For example the common value of $\mu _1 = 0.235$ for a 1 kg C tree purely characterises the size-distribution shape. The same shape of distribution would have a $\mu _r = 58.75$ for a 1000 kg C tree.

We will also define the ratio of mortality to assimilate for trees of 1 kg C size, $\mu _{p1}$, defined as follows:

19 $$\begin{aligned} \mu _{p1} = \dfrac{\gamma m_1 }{p_1} = \mu _1 (1 - \alpha ). \end{aligned}$$

To test if biomass is maximised we fix all parameters except $\alpha$, growth $g_1$, $\mu _1$ and evaluate Eq. (18) with increasing $\alpha$. In this situation we have a trade-off, as a tree of any given size has a fixed amount of assimilate to allocate to either growth or reproduction. Allocating more to growth allows the existing trees to grow larger but reduces the number of trees.

**Figure 4:**
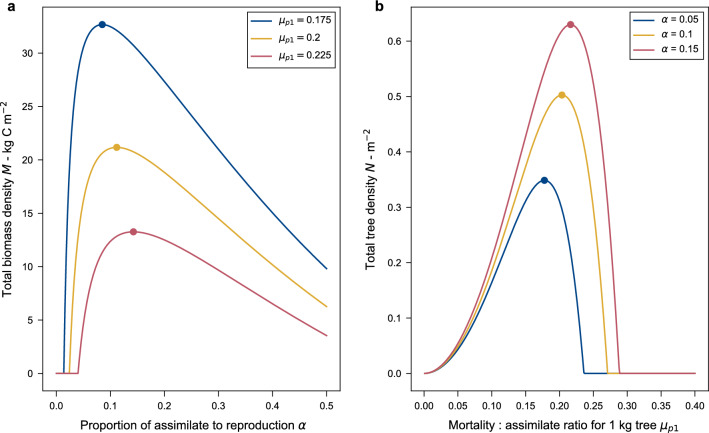
Total forest biomass and tree density optima. (**a**) Biomass as a function of the proportion of assimilate (NPP minus litter losses) allocated to reproduction. (**b**) Tree density as a function of the mortality to assimilate ratio. For both panels the parameters used are seed mass $m_s =10^{-4}$ kg C, $f_{rs} = 7\times 10^{-5}$, crown area $a_1 = 0.5$ $\hbox {m}^2$.

This leads to the forest biomass maxima seen in Fig. 4a. Where $\alpha$ is less than the peak value the biomass is limited by the number of seeds being recruited, while $\alpha$ is greater than the peak value, biomass is limited by the diminishing growth rate. The position and magnitude of the optima are altered by changing $\mu _{p1}$, seed mass, $m_s$, and $f_{rs}$ but $a_1$ only affects the magnitude of the maxima, not its position (see equations describing the optima in Supplementary material sections 4.1 and 4.2).

For tree density, we find a maximum in terms of the mortality to assimilate ratio with all other parameters held constant including $\alpha$ (Fig. 4b).

**Figure 5:**
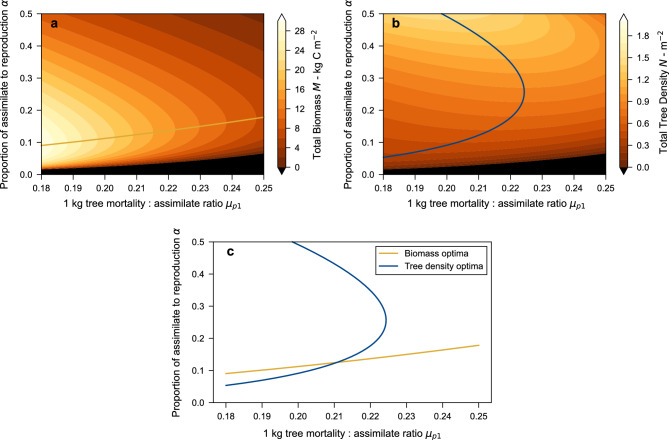
Simultaneous maxima for total forest biomass and tree density. (**a**) Biomass (contours) as a function of proportion of assimilate allocated to reproduction $\alpha$ and the mortality to assimilate ratio $\mu _{p1}$. The line shows the optimum biomass in terms of $\alpha$ for each value of $\mu _{p1}$. (**b**) Tree density (contours) in terms of $\alpha$ and $\mu _{p1}$. Line shows the optimum tree density in terms of $\mu _{p1}$ for each value of $\alpha$. (**c**) intersection of biomass and tree density optima, representing the simultaneous maxima (Demographic Optimality). For all panels the parameters used are seed mass $m_s =10^{-4}$ kg C, $f_{rs} = 7\times 10^{-5}$, crown area $a_1 = 0.5$ $\hbox {m}^2$. The black regions on panels (**a**) and (**b**) represent regions where it is not possible for forest to exist.

**Figure 6:**
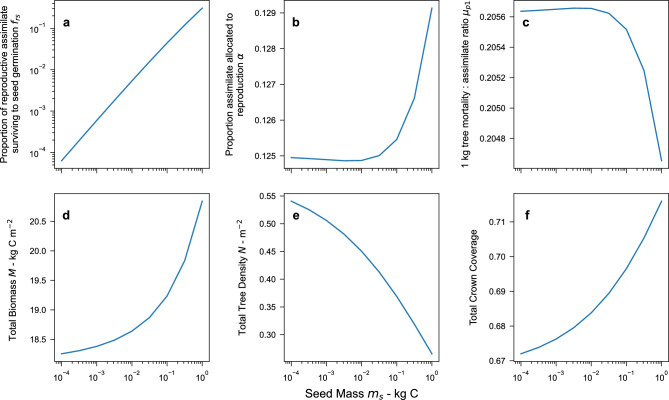
Demographic Optimality with the observed mortality to grow ratio $\mu _1 =0.235$ as a function of seed size. (**a**) fraction of reproductive carbon surviving to seed germination. The line approximates a near linear power law with exponent 0.92. (**b**) fraction of assimilate allocated to reproduction. (**c**) mortality to assimilate ratio for 1 kg C size tree. (**d**) total forest biomass density. (**e**) total forest tree density. (**f**) fractional coverage of tree crowns. For all panels the 1 kg C tree crown area $a_1= 0.5$ $\hbox {m}^2$.

For a fixed line $\alpha$, $\mu _{p1}$ is directly proportional to the mortality growth ratio, $\mu _1$. A higher value of $\mu _1$ means growth is lower relative to mortality, and for a lower value of $\mu _1$ it is relatively higher. As previously discussed, $\mu _1$ determines the shape of the size distribution, with a faster decline in tree abundance with size for higher $\mu _1$ values (Fig. 2). The amount of seeds produced is a product of the shape of the distribution, with larger trees producing more seeds, hence more seeds are produced on average per tree for lower values of $\mu _1$ (and hence $\mu _{p1}$ along a line of fixed $\alpha$). The mean crown area is also higher for low $\mu _1$, again due to the greater numbers of large trees. Initially, tree density *N* increases with increasing $\mu _{p1}$. This is because the mean crown area, $\bar{a}$, decreases faster than the effect of decreasing seed production (Eq. 17). As $\mu _{p1}$ increases further, the rate of decrease of $\bar{a}$ reduces and the effect of the falling seed production starts to dominate, so that *N* now decreases. This leads to the peak seen in tree density seen in Fig. 4b.

To see the more general picture contour plots of tree density and biomass can be shown on axes of $\alpha$ vs $\mu _{p1}$ for constant seed mass and $f_{rs}$ (Fig. 5). These plots show biomass has a maximum in terms of $\alpha$ but not $\mu _{p1}$, and the converse is true for tree density. Biomass is highest for low $\mu _{p1}$ and $\alpha$ and decreases and both increase, while tree density increases in a curving path starting low at low $\alpha$ and $\mu _{p1}$ and increasing through middling values of before starting curve back to its highest values for low $\mu _{p1}$ and high $\alpha$.

By themselves each of the optima for biomass and tree density alone suggest unrealistic values of parameters such as $\alpha$. Following along the biomass optima to higher biomass suggests a forest corresponding to the global maximum biomass would have a value of $\alpha$ that is low compared to observations and also growth very much higher than mortality, rather than the value that we widely observe. This would imply a forest of a small number of very large trees. For tree density, the global maximum would have a very high $\alpha$ (close to 1) and a low $\mu _{p1}$ (but $\mu _1$ would be high due to the large $\alpha$), suggesting an unrealistic forest of very many very small trees.

However, plotting the lines of these maxima on each plot and then together without the contours (Fig. 5c), show they cross at a particular $\alpha$ and $\mu _{p1}$. This special point has the forest simultaneously maximising its biomass in terms of its $\alpha$ and its tree density in terms of its $\mu _{p1}$. We call this intersection point “Demographic Optimality (DO)”.

### Demographic optimality and observed mortality to growth ratio

Our hypothesis is that the common shape of the demographic profiles that we see across sites in North America and Amazonia (Fig. 1), is consistent with this definition of Demographic Optimality.

We used the RAINFOR Amazon forest inventory dataset^21^ to find the mortality-to-growth ratio $\mu _1$ for a large number of forest plots across the Amazon. We derived a central estimate of $\mu _1=0.198$ for a tree with 1 kg dry tree mass. To convert to kg of carbon we assume approximately half the dry mass is carbon^22^. We must account for this factor in both the reference tree size and also on the tree growth rate, which leads to an adjustment factor $2^{1 - 3/4} = 2^{1/4} = 1.189$ (where the power is one minus the growth scaling power). Our best-fit value of $\mu _1$, in terms of kgC, is therefore 0.235 (See supplementary material section 3).

The only variable that moves the intersecting maxima in $\alpha$ - $\mu _{p1}$ space for any given seed mass, is $f_{rs}$. We can therefore use numerical methods to find the value of $f_{rs}$ that corresponds to the intersecting maxima having an $\mu _1$ of exactly 0.235. This process can be repeated for a range of seed mass values (see Fig. 6).

For any given value of seed mass, there is a corresponding DO state that gives $\mu _1=0.235$, the proportion of assimilate allocated to reproduction that ends up as seeds that survive to germination ($f_{rs}$) increases almost linearly with seed mass. A simple linear regression gives $f_{rs} \approx 0.35 m_s^{0.926}$ (see Fig. 6a) . The values of $f_{rs}$ range from 0.35 for a very large 1 kg C seed, to $6.1 \times 10^{-4}$ for 1 g C seed to $1.1 \times 10^{-6}$ for a 1 mg C seed.

This relationship suggests that smaller seeds are much less likely to end up as seedlings. The fraction of assimilate allocated to reproduction $\alpha$ remains surprisingly insensitive to changing seed mass, increasing from 12.5% to 13% of assimilate allocated to reproduction. The mortality to assimilate ratio $\mu _{p1}$ also hardly changes and gives a value of 0.205. The biomass and fractional canopy coverage increase with increasing seed mass, while the tree density declines. This suggests forests with smaller seed sizes should have smaller but more numerous trees compared to those with larger seed sizes.

## Discussion

Demographic Equilibrium Theory (DET) gives a maximum in forest biomass as a function of reproductive allocation ($\alpha$), and also a maximum in total tree number density as a function of the ratio of mortality to assimilate ratio ($\mu _{p1}$). It is possible to find an intersection point where both biomass and tree density are on their maximisation curves, a point that we have called demographic optimality (DO). The shape of Amazon and US forest size distributions have been previously observed^8,9^ to be very similar, as characterised by their mortality-to-growth rate ratio $\mu _1 = 0.235$. We have hypothesised that this common value of $\mu _1$ is consistent with DO, if the proportion of reproductive assimilate that survives through to seed germination ($f_{rs}$) has a near-linear dependence on seed size. Both of the two optima studied have, for the range of $\alpha$ we expect to see, show a positive gradient of $\alpha$ to $\mu _{p1}$. This suggests trees having lower assimilate or experiencing higher mortality will need to focus more on reproduction to have the maximum possible biomass. It also suggests that trees with higher proportion of assimilate allocated to reproduction (up to 0.25) will need to have higher growth relative to mortality to maximise tree density.

The DO model produces a near-linear relationship between the parameter $f_{rs}$ and the seed size. This suggests that smaller seeds undergo higher reproductive costs and seed mortality, consistent with studies that reconstruct the relationship of seed size to seed mortality from many previous field studies^23,24^ and to theories exploring the trade-off of different seed sizes^25^. Currently the DO model only gives us the overall reproductive cost implied by the $f_{rs}$ parameter, and does not give any indication of how the three main processes of reproductive costs, pre-dispersal and post-dispersal (seed pool) losses relate to seed size. This makes it difficult to compare to previous studies such as those by Greene^24^ and Moles^23^, as they do not include all three aspects of reproductive costs.

A further interesting result is that the model implies both the allocation to reproduction $\alpha$ and the mortality to assimilate ratio for a 1 kg C tree $\mu _{p1}$ are nearly invariant to changing seed size. The value of $\alpha$ suggested by the DO model of about 13% falls nicely within the range suggested by both Malhi^10^ (5-15% NPP) and Schaefer^11^ (3-20 % GPP). The $\mu _{p1}$ value is even more invariant to seed size with a value close to 0.205. This suggests, averaged over the large scale, that forests such as the Amazon would be expected to allocate around an eighth of its assimilate to reproduction. Similarly such a forest would have its small trees expected to have a rate of assimilate production around five times that of their mean mortality rate, with this ratio increasing with tree size. These results suggest suitable values for these parameters within DGVMs.

This study hypothesises DO as a possible explanation for the remarkable similarity between forest size distributions, in otherwise very different forests. This hypothesis is based-on Demographic Equilibrium Theory (DET), and so applies generally to forests that are not heavily managed or recently disturbed. More complex models often contain many more processes and parameters, but their equilibria will still imply effective parameters within a DET profile.

We have not discussed how the DO state may be arrived at, although competition between trees of different sizes and trees of different types, are the most likely mechanisms. For example, the mean mortality rate which is a key influence on the shape of tree-size distributions, implicitly includes the impacts of competition for space and light.

Are these predictions subject to the effects of climate change and the effect of increased growth due to $\hbox {CO}_2$ fertilization? If the common value of $\mu _1$ and the predicted $\alpha$ are robust to such changes then the biomass of the forest would likely be unaffected. The $\mu _1$ value itself does not specify absolute growth or mortality just their relative magnitudes, so it is possible for two forests to have the same $\mu _1$ but different growth rates, if mortality increases also. Recent studies^26–28^ both suggest an increase in both mortality and growth but at the moment suggest an overall reduction in the carbon sink and hence $\mu _1$ increasing overall.

## Conclusions

The number density of trees as a function of tree-size (the ‘tree-size distribution’) has been found to have a remarkably similar shape across very different forest sites in Amazonia and the USA. In a steady state, this suggests a common ratio of tree mortality-rate to tree growth-rate for a tree of a given mass. We have shown that such a common ratio of mortality to growth is consistent with the simultaneous maximisation of the tree number and total biomass of a forest, which we term ’Demographic Optimality (DO)’. If the DO state is maintained it implies that tree mortality rates will vary proportionally to tree growth-rate, and that forest biomass will be largely insensitive to $\hbox {CO}_2$ fertilization of photosynthesis.

### Supplementary Information

Supplementary Information.

## Data Availability

The python code and data used used to generate the figures in this study, using the equations from the text and supplement is available via Zenodo at https://doi.org/10.5281/zenodo.7805755.
