# Supplementary material for: A theory of demographic optimality in forests
Source: Sci Rep. 2023 Oct 31;13:18712. doi: 10.1038/s41598-023-44860-7 (PMC10618179; doi:10.1038/s41598-023-44860-7)
Supplement: Supplementary file 1 — Supplementary Information. [file 41598_2023_44860_MOESM1_ESM.pdf]

Supplementary material for A theory of demographic optimality

Jonathan R Moore

August 2023

Contents

1 Detailed proofs of deriving the equations for total forest properties 2

1.1 Total tree density . . . 2

1.2 Total forest biomass density . . . 3

2 Mean Properties and Compact DET Equations 4

3 Converting μ from dry to carbon mass 5

4 Equations for lines of optima (nullclines) 6

4.1 Biomass exact implicit equation . . . 6

4.2 Tree Density exact implicit equation . . . 7

4.3 Biomass approximate explicit equation . . . 7

5 Proof for equations of lines of optima 8

5.1 Forest Properties for Constant Tree Assimilate . . . 8

5.2 Differentiating Coverage with respect to α . . . 11

5.3 Differentiating Biomass with respect to α . . . 11

5.4 Exact Biomass Implicit Nullcline Solution . . . 13

5.5 Explicit Biomass Approximate Nullcline Solution . . . 14

5.6 Differentiating N with respect to μ_{p1}, for fixed seedling mass . 16

5.7 Exact N Implicit Nullcline Solution . . . 17

1 Detailed proofs of deriving the equations for total forest properties

Equilibrium forest properties such as total forest stem density N, total forest biomass M, total forest net growth G, coverage ν and net forest assimilate P can be obtained through integration of the equilibrium RED solution. Starting from the size distribution equation

$$n = n_r \left(\frac{m}{m_r}\right)^{-3/4} \exp\left(4\mu_r \left[1 - \left(\frac{m}{m_r}\right)^{1/4}\right]\right) \tag{1}$$

1.1 Total tree density

$$N = \int_{m_s}^{\infty} n(m)dm = n_r m_r^{3/4} \exp(4\mu_r) \int_{m_s}^{\infty} m^{-3/4} \exp\left(-4\mu_r \left(\frac{m}{m_s}\right)^{1/4}\right) dm \tag{2}$$

if we make the substitution

$$y = 4\mu_r (m/m_r)^{1/4} \tag{3}$$

and so

$$dy = \frac{\mu_r}{m_r^{1/4}} m^{-3/4} dm \tag{4}$$

leading to

$$dm = \frac{m_r^{1/4}}{\mu_r} m^{3/4} dy \tag{5}$$

substituting these equations 3 and 5 into equation 2 gives

$$N = \int_{m_s}^{\infty} n(m)dm = \frac{n_r m_r}{\mu_r} \exp(4\mu_r) \int_{4y_s}^{\infty} \exp(-y)dy \tag{6}$$

where $y_s = y(m_s) = 4\mu_r (m_s/m_r)^{1/4} = 4\mu_s$, then we get

$$N = \int_{m_s}^{\infty} n(m)dm = \frac{n_r m_r}{\mu_r} \frac{\exp(4\mu_r)}{\exp(4\mu_s)} = \frac{n_s m_s}{\mu_s} = \frac{n_s g_s}{\gamma} \tag{7}$$

1.2 Total forest biomass density

$$M = \int_{m_s}^{\infty} n(m)\, m\, dm = n_r m_r^{3/4} \exp(4\mu_r) \int_{m_s}^{\infty} m^{1/4} \exp\left(-4\mu_r \left(\frac{m}{m_r}\right)^{1/4}\right) dm \tag{8}$$

if we again substitute for y then

$$M = 4 n_r m_r^{3/4} \exp(4\mu_r) \int_{y_s}^{\infty} \frac{m^{5/4}}{y} \exp(-y) dy \tag{9}$$

If we eliminate m using equation 3

$$M = \frac{n_r m_r^2 \exp(4\mu_r)}{\mu_r (4\mu_r)^4} \int_{y_s}^{\infty} y^4 \exp(-y) dy \tag{10}$$

the integral is now a standard one for an upper incomplete Gamma function

$$M = \frac{n_r m_r^2 \exp(4\mu_r)}{\mu_r (4\mu_r)^4} \Gamma(5, 4\mu_s) \tag{11}$$

Now using the equation 7 we can say

$$M = \frac{m_r N \exp(4\mu_s)}{(4\mu_r)^4} \Gamma(5, 4\mu_s) \tag{12}$$

which using

$$\mu_s = \frac{\gamma m_s}{g_s} = \frac{\gamma m_s}{g_r (\frac{m_s}{m_r})^{3/4}} = \mu_r \left(\frac{m_s}{m_r}\right)^{1/4} \tag{13}$$

finally becomes

$$M = \frac{m_s N \exp(4\mu_s)}{(4\mu_s)^4} \Gamma(5, 4\mu_s) \tag{14}$$

The properties of the upper incomplete Gamma function are such that when the first parameter is an integer it will evaluate to a finite series, which in this case is

$$M = \int_{m_s}^{\infty} n(m)\, m\, dm = m_s\, N \left(1 + \frac{1}{\mu_s} + \frac{3}{4\mu_s^2} + \frac{3}{8\mu_s^3} + \frac{3}{32\mu_s^4}\right) \tag{15}$$

Similarly we can derive the coverage

$$\nu = \int_{m_s}^{\infty} n(m)\, a(m)\, dm = a_s N \left(1 + \frac{1}{2\mu_s} + \frac{1}{8\mu_s^2}\right) \tag{16}$$

where $a(m)$ is the tree crown area as a function of tree carbon mass $a(m) = a_r\left(\frac{m}{m_r}\right)^{1/2}$ and $a_s = a(m = m_s)$. The total forest growth is

$$G = \int_{m_s}^{\infty} n(m)\, g(m)\, dm = g_s N_s \left(1 + \frac{3}{4\mu_s} + \frac{3}{8\mu_s^2} + \frac{3}{32\mu_s^3}\right) \tag{17}$$

where $g(m) = g_r\left(\frac{m}{m_r}\right)^{3/4}$ and $g_s = g(m = m_s)$. The total forest assimilate is

$$P = \int_{m_s}^{\infty} n(m)\, p(m)\, dm = p_s N_s \left(1 + \frac{3}{4\mu_s} + \frac{3}{8\mu_s^2} + \frac{3}{32\mu_s^3}\right) \tag{18}$$

where $p(m) = p_r\left(\frac{m}{m_r}\right)^{3/4}$ and $p_s = p(m = m_s)$.
If the first Gamma function parameter is not an integer then the solution to each of these equations remains as a purely an upper incomplete Gamma function. Luckily, the MST allometry results in more convenient short finite series, which is much easier to use.

2 Mean Properties and Compact DET Equations

We can rewrite DET in terms of the mean gridbox forest properties. These tell us what the mean tree would be like if the forest had identical trees rather than the size distribution. These mean properties do not depend on the final closed-form solution or the number of trees, but are purely a function of tree traits γ, g_s, a_s and m_s.

$$\bar{g} = \frac{G}{N} = g_s\Big(1 + \frac{3}{4\mu_s} + \frac{3}{8\mu_s^2} + \frac{3}{32\mu_s^3}\Big) \tag{19}$$

$$\bar{m} = \frac{M}{N} = m_s\Big(1 + \frac{1}{\mu_s} + \frac{3}{4\mu_s^2} + \frac{3}{8\mu_s^3} + \frac{3}{32\mu_s^4}\Big) \tag{20}$$

$$\bar{a} = \frac{\nu}{N} = a_s\Big(1 + \frac{1}{2\mu_s} + \frac{1}{8\mu_s^2}\Big) \tag{21}$$

$$\bar{p} = \frac{P}{N} = p_s\Big(1 + \frac{3}{4\mu_s} + \frac{3}{8\mu_s^2} + \frac{3}{32\mu_s^3}\Big) \tag{22}$$

We can also define a mean tree seed production rate $\bar{s}$

$$\bar{s} = \alpha\frac{P}{N m_s} = \alpha\frac{\bar{p}}{m_s} = \frac{\alpha p_s}{m_s}\Big(1 + \frac{3}{4\mu_s} + \frac{3}{8\mu_s^2} + \frac{3}{32\mu_s^3}\Big) \tag{23}$$

So now

$$M = N\bar{m} \tag{24}$$

$$G = N\bar{g} \tag{25}$$

$$\nu = N\bar{a} \tag{26}$$

3 Converting μ from dry to carbon mass

Define m as carbon mass of a tree and m_d as dry mass of the same tree. If we assume the carbon content of the tree is approximately half its dry mass then can assume $m_d = 2m$.
Growth rate of the tree in terms of carbon mass g in terms of growth g_r at reference tree mass m_r is

$$g(m) = g_r(m/m_r)^{\phi} \tag{27}$$

and for dry mass the growth rate g_d is

$$g_d(m_d) = g_{dr}(m_d/m_{dr})^{\phi} \tag{28}$$

The growth rate of carbon mass in a tree of dry mass m_d must also be half that of the dry mass growth rate, so

$$g_d(m_d) = 2g(m = m_d/2) \tag{29}$$

so then substituting in equations 27 and 28 gives

$$\frac{g_{dr}}{m_{dr}^{\phi}} m_d^{\phi} = 2\frac{g_r}{m_r^{\phi}} m^{\phi} \tag{30}$$

the carbon mass m term on the RHS is equal to half the dry mass m_d and so

$$\frac{g_{dr}}{m_{dr}^{\phi}} m_d^{\phi} = 2^{1-\phi}\frac{g_r}{m_r^{\phi}} m_d^{\phi} \tag{31}$$

and then we can cancel the m_d terms

$$\frac{g_{dr}}{m_{dr}^{\phi}} = 2^{1-\phi}\frac{g_r}{m_r^{\phi}} \tag{32}$$

For μ we can do the same analysis and define a μ for carbon mass

$$\mu(m) = \frac{\gamma m}{g(m)} = \frac{\gamma m^{1-\phi} m_r^{\phi}}{g_r} \tag{33}$$

and dry mass

$$\mu_d(m_d) = \frac{\gamma m_d}{g_d(m_d)} = \frac{\gamma m_d^{1-\phi} m_{dr}^{\phi}}{g_{dr}} \tag{34}$$

The μ value for a tree of 1 kg of dry mass is

$$\mu_{d1} = \mu_d(m_d = 1) = \frac{\gamma m_{dr}^{\phi}}{g_{dr}} \tag{35}$$

subbing in from equation 32, allows us to relate it to μ for carbon mass

$$\mu_{d1} = \frac{\gamma m_r^{\phi}}{g_r} \frac{1}{2^{1-\phi}} = \frac{\mu(m = 1)}{2^{1-\phi}} \tag{36}$$

So finally

$$\mu_1 = 2^{1-\phi} \mu_{d1} \tag{37}$$

For RAINFOR we have $\mu_{d1} = 0.198$ and $\phi = 0.75$ so we get $\mu_1 = 0.235$

4 Equations for lines of optima (nullclines)

Easier to write the optima more compactly using a new variable z

$$z = 4\mu_{pr} \left(\frac{m_s}{m_r} \right)^{1/4} = 4\mu_s(1 - \alpha) \tag{38}$$

4.1 Biomass exact implicit equation

This equation exactly describes the optima of biomass with respect to α.

$$
\begin{aligned}
&z^{13} + z^{12}\,(9 - 10\alpha) + 2z^{11}\,\big((25 - 4f_{rs}))\alpha^2 - 48\alpha + 23\big) \\
&- 4z^{10}\,(\alpha - 1)\,\big(8(5 - 4f_{rs})\alpha^2 - 81\alpha + 41\big) \\
&- 8z^{9}\,(\alpha - 1)^2\,\big((125f_{rs} - 48)\alpha^2 + 102\alpha - 54\big) \\
&+ 12z^{8}\,(\alpha - 1)^3\,\big(2(206f_{rs} - 33)\alpha^2 + 137\alpha - 71\big) \\
&- 96z^{7}\,(\alpha - 1)^4\,\big((179f_{rs} - 14)\alpha^2 + 27\alpha - 13\big) \\
&+ 144z^{6}\,(\alpha - 1)^5\,\big(4(77f_{rs} - 3)\alpha^2 + 21\alpha - 9\big) \\
&- 288z^{5}\,(\alpha - 1)^6\,\big((301f_{rs} - 5)\alpha^2 + 8\alpha - 3\big) \\
&+ 288z^{4}\,(\alpha - 1)^7\,\big((446f_{rs} - 2)\alpha^2 + 3\alpha - 1\big) \\
&- 141696\alpha^2 f_{rs} z^3\,(\alpha - 1)^8 \\
&+ 110592\alpha^2 f_{rs} z^2\,(\alpha - 1)^9 \\
&- 55296\alpha^2 f_{rs} z\,(\alpha - 1)^{10} \\
&+ 13824\alpha^2 f_{rs}\,(\alpha - 1)^{11} = 0
\end{aligned}
\tag{39}
$$

4.2 Tree Density exact implicit equation

$$
\begin{aligned}
&- z^9 - 10(1-\alpha)z^8 + 2(1-\alpha)(4\alpha f_{rs} - 21(1-\alpha))z^7 \\
&+ 32(1-\alpha)^2(2\alpha f_{rs} - 3(1-\alpha))z^6 \\
&+ 24(1-\alpha)^3(11\alpha f_{rs} - 5(1-\alpha))z^5 \\
&+ 72(1-\alpha)^4(10\alpha f_{rs} + \alpha - 1)z^4 + 1344\alpha f_{rs}(1-\alpha)^5 z^3 \\
&+ 1728\alpha f_{rs}(1-\alpha)^6 z^2 + 1440\alpha f_{rs}(1-\alpha)^7 z + 576\alpha f_{rs}(1-\alpha)^8 = 0
\end{aligned}
\tag{40}
$$

4.3 Biomass approximate explicit equation

We can also write the biomass optima approximately but in explicit form

$$
\alpha \approx \frac{4\mu_{pr}^2 m_s^{1/2}}{\sqrt{m_r f_{rs}\left(12\mu_{pr}\left(\frac{m_s}{m_r}\right)^{1/4} + 3\right)}}
\tag{41}
$$

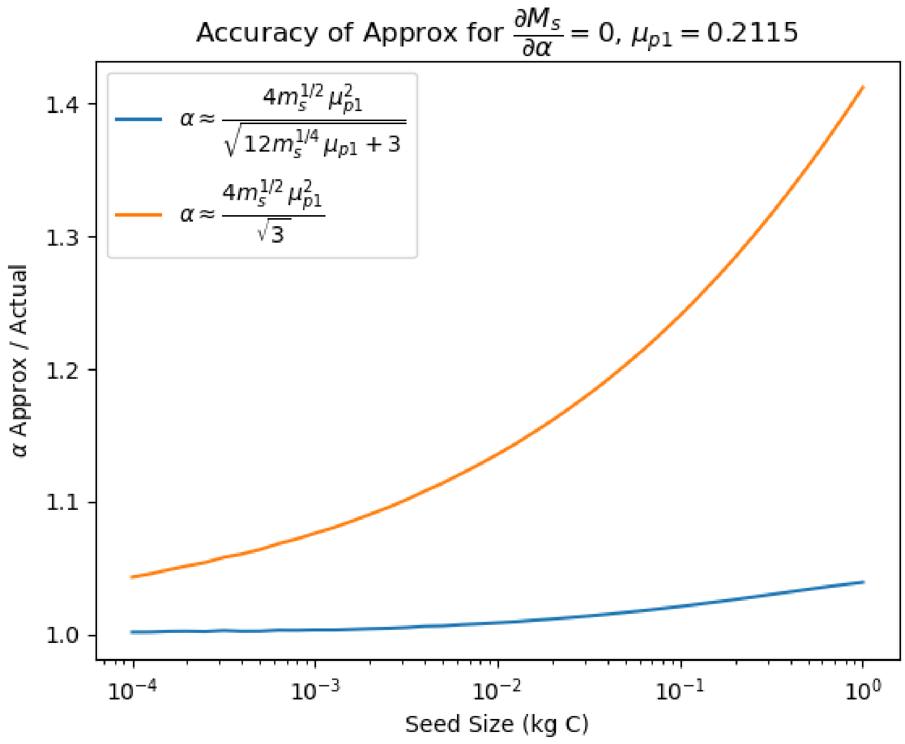

Figure 1: Shows the accuracy of the approximate solutions for the biomass nullcline compared to the exact solution. The more complex solution gives very accurate results even for large seed sizes.

5 Proof for equations of lines of optima

5.1 Forest Properties for Constant Tree Assimilate

To study the effect of optimum seed allocation fraction α it is useful to assume the assimilate p_r is constant. This means if α varies then there is trade-off between seed production and growth. It is also useful to study how varying the seed mass m_s may affect the results. To do this it is necessary to more explicitly show which terms have dependence on α and m_s.

As the assimilate rate p_r is fixed we need replace μ with an equivalent in terms of assimilate, this will be the mortality to assimilate ratio μ_p. For a fixed reference mass m_r, this is defined as

$$\mu_{pr} = \frac{\gamma m_r}{p_r} \tag{42}$$

where p_r is the net assimilate for a tree of size m_r. As growth and assim-

ilate are related by $g = p(1-\alpha)$ we can similarly relate μ_r and μ_{pr}

$$\mu_{pr} = (1-\alpha)\frac{\gamma m_r}{g_r} = (1-\alpha)\mu_r \tag{43}$$

So, μ_s can now be written in terms of μ_{pr} as a function of α and m_s

$$\mu_s = \frac{\mu_{ps}}{1-\alpha} = \mu_r\left(\frac{m_s}{m_r}\right)^{1/4} = \frac{\mu_{pr}}{1-\alpha}\left(\frac{m_s}{m_r}\right)^{1/4} \tag{44}$$

This is important as it allows the parts that remains constant (μ_{pr} and m_r) to be isolated from both α and m_s, which will vary. So, μ_s is a function of both α and m_s.

To write the DET equations more compactly we use a new variable z, which only varies with m_s (doesn't vary with α)

$$z = 4\mu_{pr}\left(\frac{m_s}{m_r}\right)^{1/4} = 4\mu_s(1-\alpha) \tag{45}$$

so

$$\mu_s = \frac{z}{4(1-\alpha)} \tag{46}$$

we can then use this to rewrite the mean forest properties in a form that separates out the α dependence

$$\bar{g} = g_s\Big(\frac{z^3 + 3z^2(1-\alpha) + 6z(1-\alpha)^2 + 6(1-\alpha)^3}{z^3}\Big) \tag{47}$$

$$\bar{m} = m_s\Big(\frac{z^4 + 4z^3(1-\alpha) + 12z^2(1-\alpha)^2 + 24z(1-\alpha)^3 + 24(1-\alpha)^4}{z^4}\Big) \tag{48}$$

$$\bar{a} = a_s\Big(\frac{z^2 + 2z(1-\alpha) + 2(1-\alpha)^2}{z^2}\Big) \tag{49}$$

$$\bar{p} = p_s\Big(\frac{z^3 + 3z^2(1-\alpha) + 6z(1-\alpha)^2 + 6(1-\alpha)^3}{z^3}\Big) \tag{50}$$

$$\bar{s} = \alpha\frac{\bar{p}}{m_s} = \frac{\alpha p_s}{m_s}\Big(\frac{z^3 + 3z^2(1-\alpha) + 6z(1-\alpha)^2 + 6(1-\alpha)^3}{z^3}\Big) \tag{51}$$

We can then make the equations more compact by making the substitutions

$$Z_G = z^3 + 3z^2(1-\alpha) + 6z(1-\alpha)^2 + 6(1-\alpha)^3 \tag{52}$$

$$Z_M = z^4 + 4z^3(1-\alpha) + 12z^2(1-\alpha)^2 + 24z(1-\alpha)^3 + 24(1-\alpha)^4 \tag{53}$$

$$Z_\nu = z^2 + 2z(1-\alpha) + 2(1-\alpha)^2 \tag{54}$$

Note these expressions are related

$$Z_G = z^3 + 3Z_\nu(1-\alpha) \tag{55}$$

$$Z_M = z^4 + 4Z_G(1-\alpha) = z^4 + 4z^3(1-\alpha) + 12Z_\nu(1-\alpha)^2 \tag{56}$$

then the mean forest properties are

$$\bar{g} = g_s\left(\frac{Z_G}{z^3}\right) \tag{57}$$

$$\bar{m} = m_s\left(\frac{Z_M}{z^4}\right) \tag{58}$$

$$\bar{a} = a_s\left(\frac{Z_\nu}{z^2}\right) \tag{59}$$

$$\bar{p} = p_s\left(\frac{Z_G}{z^3}\right) \tag{60}$$

$$\bar{s} = \frac{\alpha p_s}{m_s}\left(\frac{Z_G}{z^3}\right) = \alpha\gamma\left(\frac{Z_G}{z^4}\right) \tag{61}$$

So now we can rewrite the coverage equation

$$\nu = 1 - \frac{z^4}{4\alpha Z_G} \tag{62}$$

and

$$M = \frac{m_s}{a_s}\frac{Z_M}{Z_\nu}\frac{\nu}{z^2} \tag{63}$$

5.2 Differentiating Coverage with respect to α

$$z = 4\mu_{pr}\left(\frac{m_s}{m_r}\right)^{1/4} = 4\mu_s(1-\alpha) \tag{64}$$

$$\nu = 1 - \frac{z^4}{4f_{rs}\alpha Z_G} \tag{65}$$

Coverage (equation 62) can be differentiated fairly easily, the first step leads to

$$\frac{\partial\nu}{\partial\alpha} = -\frac{z^4}{4f_{rs}}\frac{\partial}{\partial\alpha}\left(\frac{1}{\alpha Z_G}\right) = -\frac{z^4}{4f_{rs}\alpha Z_G}\left(-\frac{1}{\alpha}\frac{\partial\alpha}{\partial\alpha} - \frac{1}{Z_G}\frac{\partial Z_G}{\partial\alpha}\right) \tag{66}$$

$$\frac{\partial\nu}{\partial\alpha} = (1-\nu)\left(\frac{1}{\alpha} + \frac{1}{Z_G}\frac{\partial Z_G}{\partial\alpha}\right) \tag{67}$$

noting that

$$\frac{\partial Z_G}{\partial\alpha} = -z^2 - 12z(1-\alpha) - 18(1-\alpha)^2 = \frac{(Z_G - z^3)z - 3Z_G(1-\alpha)}{(1-\alpha)^2} \tag{68}$$

then

$$\frac{\partial\nu}{\partial\alpha} = \frac{(1-\nu)}{(1-\alpha)^2}\left(\frac{1}{\alpha} + z - 1 - 4(1-\alpha) - \frac{z^4}{Z_G}\right) \tag{69}$$

finally we move denominator terms outside of the brackets

$$\frac{\partial\nu}{\partial\alpha} = \frac{(1-\nu)}{Z_G\alpha(1-\alpha)^2}\left(Z_G + \alpha\Big[Z_G\Big\{z - 1 - 4(1-\alpha)\Big\} - z^4\Big]\right) \tag{70}$$

5.3 Differentiating Biomass with respect to α

Starting with the biomass equation

$$M = \frac{m_s}{a_s}\frac{Z_M}{Z_\nu}\frac{\nu}{z^2} \tag{71}$$

we can then differentiate it by using a modified version of the chain rule

$$\frac{\partial M}{\partial\alpha} = M\left(\frac{1}{Z_M}\frac{\partial Z_M}{\partial\alpha} + \frac{1}{\nu}\frac{\partial\nu}{\partial\alpha} - \frac{1}{Z_\nu}\frac{\partial Z_\nu}{\partial\alpha}\right) \tag{72}$$

we can then substitute in the equations for $\frac{\partial Z_M}{\partial\alpha}$ and $\frac{\partial Z_\nu}{\partial\alpha}$

$$\frac{\partial Z_M}{\partial \alpha} = -4z^3 - 24z^2(1-\alpha) - 72z(1-\alpha)^2 - 96(1-\alpha)^3 = \frac{(Z_M - z^4)z - 4Z_M(1-\alpha)}{(1-\alpha)^2} \tag{73}$$

$$\frac{\partial Z_\nu}{\partial \alpha} = -2z - 4(1-\alpha) = \frac{(Z_\nu - z^2)z - 2Z_\nu(1-\alpha)}{(1-\alpha)^2} \tag{74}$$

giving

$$\frac{\partial M}{\partial \alpha} = M\left(\frac{(Z_M - z^4)z - 4Z_M(1-\alpha)}{Z_M(1-\alpha)^2} + \frac{1}{\nu}\frac{\partial \nu}{\partial \alpha} - \frac{(Z_\nu - z^2)z - 2Z_\nu(1-\alpha)}{Z_\nu(1-\alpha)^2}\right) \tag{75}$$

and then substitute in the coverage differential (equation 70) to get the biomass differential

$$\boxed{\begin{aligned}\frac{\partial M}{\partial \alpha} = \frac{M}{(1-\alpha)^2}\Bigg[&\frac{(Z_M - z^4)z}{Z_M} \\ &+ \frac{(1-\nu)}{\nu}\left(\frac{1}{\alpha} + z - 1 - 4(1-\alpha) - \frac{z^4}{Z_G}\right) \\ &- \frac{(Z_\nu - z^2)z}{Z_\nu} - 2(1-\alpha)\Bigg]\end{aligned}} \tag{76}$$

To solve the case where $\frac{\partial M}{\partial \alpha} = 0$ it is helpful to get rid of the fractions inside the square brackets. First, tidy up the coverage differential

$$\begin{aligned}\frac{\partial M}{\partial \alpha} = \frac{M}{(1-\alpha)^2}\Bigg[&\frac{(Z_M - z^4)z}{Z_M} \\ &+ \frac{(1-\nu)}{Z_G\alpha\nu}\left(Z_G + \alpha\Big[Z_G\Big\{z - 1 - 4(1-\alpha)\Big\} - z^4\Big]\right) \\ &- \frac{(Z_\nu - z^2)z}{Z_\nu} - 2(1-\alpha)\Bigg]\end{aligned} \tag{77}$$

then move the denominators outside the square brackets

$$\begin{aligned}\frac{\partial M}{\partial \alpha} = \frac{M}{Z_M Z_\nu Z_G \alpha\nu(1-\alpha)^2}\Bigg[& \\ Z_\nu Z_M(1-\nu)&\left(Z_G + \alpha\Big[Z_G\Big\{z - 1 - 4(1-\alpha)\Big\} - z^4\Big]\right) \\ + Z_G\alpha\nu&\Big(Z_\nu(Z_M - z^4)z - Z_M(Z_\nu - z^2)z - 2Z_M Z_\nu(1-\alpha)\Big)\Bigg]\end{aligned} \tag{78}$$

Then replace the $1-\nu$ term using equation 62 and move the denominator outside the brackets

$$
\begin{aligned}
\frac{\partial M}{\partial \alpha} = \frac{M}{4Z_M Z_\nu Z_G^2 f_{rs}\alpha^2\nu(1-\alpha)^2} \Bigg[\\
Z_\nu Z_M z^4 \left(Z_G + \alpha \Big[Z_G \{ z - 1 - 4(1-\alpha) \} - z^4 \Big] \right) \\
+ Z_G \alpha \Big(4\alpha f_{rs} Z_G - z^4 \Big) \Big(Z_\nu (Z_M - z^4) z - Z_M (Z_\nu - z^2) z - 2 Z_M Z_\nu (1-\alpha) \Big) \Bigg]
\end{aligned} \tag{79}
$$

From this it is possible to obtain both an exact implicit solution to the nullcline or an explicit approximation.

To find the nullcline we look for where

$$\frac{\partial M}{\partial \alpha} = 0 \tag{80}$$

which as we only interested in cases where $M > 0$, this implies

$$
\begin{aligned}
Z_\nu Z_M z^4 \left(Z_G + \alpha \Big[Z_G \{ z - 1 - 4(1-\alpha) \} - z^4 \Big] \right) \\
+ Z_G \alpha \Big(4\alpha f_{rs} Z_G - z^4 \Big) \Big(z^3 (Z_M - Z_\nu z^2) - 2 Z_M Z_\nu (1-\alpha) \Big) = 0
\end{aligned} \tag{81}
$$

5.4 Exact Biomass Implicit Nullcline Solution

By expanding equation 81 using sympy we can obtain the exact solution as an implicit equation

$$
\begin{aligned}
&z^{13} + z^{12}(9-10\alpha) + 2z^{11}\big((25-4f_{rs}))\alpha^2 - 48\alpha + 23\big) \\
&- 4z^{10}(\alpha-1)\big(8(5-4f_{rs})\alpha^2 - 81\alpha + 41\big) \\
&- 8z^9(\alpha-1)^2\big((125f_{rs}-48)\alpha^2 + 102\alpha - 54\big) \\
&+ 12z^8(\alpha-1)^3\big(2(206f_{rs}-33)\alpha^2 + 137\alpha - 71\big) \\
&- 96z^7(\alpha-1)^4\big((179f_{rs}-14)\alpha^2 + 27\alpha - 13\big) \\
&+ 144z^6(\alpha-1)^5\big(4(77f_{rs}-3)\alpha^2 + 21\alpha - 9\big) \\
&- 288z^5(\alpha-1)^6\big((301f_{rs}-5)\alpha^2 + 8\alpha - 3\big) \\
&+ 288z^4(\alpha-1)^7\big((446f_{rs}-2)\alpha^2 + 3\alpha - 1\big) \\
&- 141696\alpha^2 f_{rs} z^3(\alpha-1)^8 \\
&+ 110592\alpha^2 f_{rs} z^2(\alpha-1)^9 \\
&- 55296\alpha^2 f_{rs} z(\alpha-1)^{10} \\
&+ 13824\alpha^2 f_{rs}(\alpha-1)^{11} = 0
\end{aligned} \tag{82}
$$

This is precise but unwieldy and also implicit, so we will approximate this equation with an explicit expression for α.

5.5 Explicit Biomass Approximate Nullcline Solution

To get an approximate solution we note that the for the nullcline $\alpha \to 0$ as $z \to 0$. Knowing this means the equation can be approximated when z is small.

First, approximation is to say that in equation 81 as typically $\alpha << 1$ then can say $1 - \alpha \approx 1$

$$
\begin{aligned}
&Z_\nu Z_M z^4\Big(Z_G + \alpha\Big[Z_G\big\{z-5\big\} - z^4\Big]\Big) \\
&+ Z_G\alpha\Big(4\alpha f_{rs} Z_G - z^4\Big)\Big(z^3(Z_M - Z_\nu z^2) - 2Z_M Z_\nu\Big) = 0
\end{aligned} \tag{83}
$$

then collect in terms of powers of α

$$
\begin{aligned}
&Z_\nu Z_M Z_G z^4 + \\
&\alpha z^4\Big[Z_\nu Z_M\left(Z_G\big\{z-5\big\} - z^4\right) - Z_G\Big(Z_M z^3 - Z_\nu z^5 - 2Z_M Z_\nu\Big)\Big] \\
&+ \alpha^2 4 f_{rs} Z_G^2\Big(Z_M z^3 - Z_\nu z^5 - 2Z_M Z_\nu\Big) = 0
\end{aligned} \tag{84}
$$

This can then be solved using the quadratic formula

$$\alpha = \frac{-B \pm \sqrt{B^2 - 4AC}}{2A} \tag{85}$$

but as within the domain of z ($0 < z < 1$) then $4AC >> B^2$ and $\sqrt{4AC} >> -B$

$$\alpha \approx \frac{\sqrt{-4AC}}{2A} = \sqrt{\frac{-C}{A}} = \sqrt{-\frac{Z_\nu Z_M z^4}{4 f_{rs} Z_G \Big(Z_M z^3 - Z_\nu z^5 - 2 Z_M Z_\nu \Big)}} \tag{86}$$

The terms Z_ν, Z_G and Z_M can be approximated by assuming both $1-\alpha \approx 1$ and that z is small enough that we can ignore terms of z^2 and also all of higher order terms of z. So

$$Z_\nu = z^2 + 2z(1-\alpha) + 2(1-\alpha)^2 \approx 2(z+1) \tag{87}$$

$$Z_G = z^3 + 3z^2(1-\alpha) + 6z(1-\alpha)^2 + 6(1-\alpha)^3 \approx 6(z+1) \tag{88}$$

$$Z_M = z^4 + 4z^3(1-\alpha) + 12z^2(1-\alpha)^2 + 24z(1-\alpha)^3 + 24(1-\alpha)^4 \approx 24(z+1) \tag{89}$$

This means we can simplify equation 86

$$\alpha \approx \sqrt{-\frac{2(z+1)z^4}{f_{rs}\Big(24(z+1)z^3 - 2(z+1)z^5 - 96(z+1)^2\Big)}} \tag{90}$$

and then the final solution is

$$\boxed{\alpha \approx \sqrt{\frac{z^4}{f_{rs}(z^5 - 12z^3 + 48z + 48)}}} \tag{91}$$

Testing with various numbers of terms in the denominator finds that excluding terms higher order than the linear z term works best over a large range of z

$$\boxed{\alpha \approx \sqrt{\frac{z^4}{48 f_{rs}(z+1)}}} \tag{92}$$

$$\boxed{\alpha \approx \frac{4\mu_{pr}^2 m_s^{1/2}}{\sqrt{m_r f_{rs}\left(12\mu_{pr}\left(\frac{m_s}{m_r}\right)^{1/4}+3\right)}}} \tag{93}$$

We can also find the optimum allocation to seeds αf_{rs}

$$\alpha f_{rs} \approx \frac{4\mu_{pr}^2 m_s^{1/2}}{\sqrt{\frac{m_r}{f_{rs}}\left(12\mu_{pr}\left(\frac{m_s}{m_r}\right)^{1/4}+3\right)}} \tag{94}$$

5.6 Differentiating N with respect to μ_{p1}, for fixed seedling mass

$$N = \frac{\nu}{\bar{a}} = \frac{\nu z^2}{a_s Z_\nu} \tag{95}$$

If we assume that seed mass is fixed m_s then a_s is also constant and can be left out of the differentiation, so now the differential is

$$\frac{\partial N}{\partial z} = N\left(\frac{1}{\nu}\frac{\partial \nu}{\partial z} + \frac{1}{z^2}\frac{\partial z^2}{\partial z} - \frac{1}{Z_\nu}\frac{\partial Z_\nu}{\partial z}\right) \tag{96}$$

$$\frac{\partial N}{\partial z} = N\left(\frac{\nu-1}{\nu z Z_G}(3zZ_\nu - 4Z_G) + \frac{2}{z} - \frac{2(z+1-\alpha)}{Z_\nu}\right) \tag{97}$$

Can simplify by defining $B = 4\alpha f_{rs} Z_G$ and substituting into ν so

$$\nu = 1 - \frac{z^4}{B} = \frac{B - z^4}{B} \tag{98}$$

and then substituting into equation 97

$$\frac{\partial N}{\partial z} = N\left(\frac{z^4}{(B-z^4)zZ_G}(3zZ_\nu - 4Z_G) + \frac{2}{z} - \frac{2(z+1-\alpha)}{Z_\nu}\right) \tag{99}$$

to get a simpler numerator then multiply by $(B - z^4)Z_G Z_\nu z$

$$\frac{\partial N}{\partial z} = \frac{N}{(B-z^4)Z_G Z_\nu z}\left(z^4 Z_\nu(3zZ_\nu - 4Z_G) + 2(B-z^4)Z_G\left[Z_\nu - z(z+1-\alpha)\right]\right) \tag{100}$$

So

$$\frac{\partial N}{\partial \mu_{p1}} = \frac{\partial N}{\partial z}\frac{\partial z}{\partial \mu_{p1}} = \frac{\partial N}{\partial z}\frac{z}{\partial \mu_{p1}} \tag{101}$$

To find the nullcline we look for where

$$\frac{\partial N}{\partial \mu_{p1}} = 0 \tag{102}$$

but as z and μ_{p1} are non-zero then the nullcline is also then defined by

$$\frac{\partial N}{\partial z} = 0 \tag{103}$$

So

$$z^4 Z_\nu (3zZ_\nu - 4Z_G) + 2(B - z^4) Z_G \left[Z_\nu - z(z + 1 - \alpha)\right] = 0 \tag{104}$$

5.7 Exact N Implicit Nullcline Solution

$$\begin{aligned}
&- z^9 - 10(1-\alpha)z^8 + 2(1-\alpha)(4\alpha f_{rs} - 21(1-\alpha))z^7 \\
&+ 32(1-\alpha)^2(2\alpha f_{rs} - 3(1-\alpha))z^6 \\
&+ 24(1-\alpha)^3(11\alpha f_{rs} - 5(1-\alpha))z^5 \\
&+ 72(1-\alpha)^4(10\alpha f_{rs} + \alpha - 1)z^4 + 1344\alpha f_{rs}(1-\alpha)^5 z^3 \\
&+ 1728\alpha f_{rs}(1-\alpha)^6 z^2 + 1440\alpha f_{rs}(1-\alpha)^7 z + 576\alpha f_{rs}(1-\alpha)^8 = 0
\end{aligned} \tag{105}$$
